# The molecular nature of the 17β-Estradiol binding site in the voltage- and Ca^2+^-activated K^+^ (BK) channel β1 subunit

**DOI:** 10.1038/s41598-019-45942-1

**Published:** 2019-07-10

**Authors:** Sara T. Granados, Karen Castillo, Felipe Bravo-Moraga, Romina V. Sepúlveda, Willy Carrasquel-Ursulaez, Maximiliano Rojas, Emerson Carmona, Yenisleidy Lorenzo-Ceballos, Fernando González-Nilo, Carlos González, Ramón Latorre, Yolima P. Torres

**Affiliations:** 1 Departamento de Nutrición y Bioquímica, Facultad de Ciencias, Pontificia Universidad Javeriana, Bogotá, Colombia; 2 Centro Interdisciplinario de Neurociencia de Valparaíso, Facultad de Ciencias, Universidad de Valparaíso, Valparaíso, Chile; 3 Center for Bioinformatics and Integrative Biology, Facultad de Ciencias de la Vida, Universidad Andrés, Bello, Chile

**Keywords:** Mechanism of action, Membrane structure and assembly, Steroid hormones, Potassium channels

## Abstract

The accessory β1 subunit modulates the Ca^2+^- and voltage-activated K^+^ (BK) channel gating properties mainly by increasing its apparent Ca^2+^ sensitivity. β1 plays an important role in the modulation of arterial tone and blood pressure by vascular smooth muscle cells (SMCs). 17β-estradiol (E2) increases the BK channel open probability *(P*_*o*_) in SMCs, through a β1 subunit-dependent modulatory effect. Here, using molecular modeling, bioinformatics, mutagenesis, and electrophysiology, we identify a cluster of hydrophobic residues in the second transmembrane domain of the β1 subunit, including the residues W163 and F166, as the binding site for E2. We further show that the increase in *P*_*o*_ induced by E2 is associated with a stabilization of the voltage sensor in its active configuration and an increase in the coupling between the voltage sensor activation and pore opening. Since β1 is a key molecular player in vasoregulation, the findings reported here are of importance in the design of novel drugs able to modulate BK channels.

## Introduction

The large conductance, Ca^2+^- and voltage-activated K^+^ (BK) channel is a tetrameric transmembrane protein composed of α subunits that form the K^+^ selective pore^1,2^. The α subunit is broadly expressed in mammalian tissues^3^, where it is co-expressed with an accessory β subunits in a tissue-specific manner^4,5^. The α subunit comprises seven transmembrane segments (S0-S6), leaving the amino terminus exposed to the external medium. The large intracellular carboxyl terminus domain consisting of two RCK domains contains the Ca^2+^ binding sites. The different β subunits (β1–β4) are formed by two transmembrane segments linked by a large extracellular loop and are responsible for the modulation of the biophysical and pharmacological characteristics of the α subunit^4,6^. The β1 subunit is abundantly expressed in smooth muscle cells and acts to increase the apparent sensitivity to Ca^2+^ and dramatically slows down the activation and deactivation channel-gating kinetics^7–12^. The presence of the BK α/β1 channel lowers the risk of pathologies associated with the vascular tone by causing a membrane hyperpolarization, decreasing vasoconstriction, and inducing a faster relaxation of the blood vessels^13,14^. β subunits appear to regulate the BK channel activity by targeting specific gating mechanisms^11,14,15^. In particular, by biasing the equilibrium resting-active of the voltage sensor towards its active configuration, β1 increases the apparent BK Ca^2+^ sensitivity^14,16^ Co-expression of BK α and β subunits can dramatically modify pharmacological responses of the channel^17–19^ as potential targets for channel modulators such as alcohol^20^, estrogens^19^, hormones^21^ and fatty acids^22,23^.

17β-Estradiol (E2) is the main circulating oestrogen in women and reaches a plasma concentration of 30–400 pg/mL before menopause. E2 regulates growth and the development of the reproductive system, also, helps to maintain the osseous tissue, the central nervous system and the vasodilatation in the vascular tissue^24^. The protective effect of E2 in the vasculature and against cardiovascular disease (CVD) has been demonstrated in several hormone replacement studies^25,26^. E2 activates BK channels^19,27–30^ via a process that requires the presence of the β1 subunit^19,31^. Valverde *et al*.^19^ were the first to propose that E2 affected BK channels by binding to β1, but it is still a matter of debate whether the agonistic action of E2 on BK channels is caused by its binding to the β1 subunit or to the α/β1 complex. Moreover, the molecular nature of the E2 binding site and the mode of action of the hormone are at present unknown.

Importantly, evidence has mounted that under physiological conditions, acute application of E2 (100 nM) decreases smooth muscle excitability by activating BK channels^29,32,33^. Notably, E2 or its membrane-impermeant form (E2-BSA) can induce a fast increase in BK channel activity in MCF-7 breast epithelial cancer cells with an EC_50_ of 80 pM reaching a maximal effect at 10 nM^34^. Rapid effects of E2 have also been reported in neurons of the area postrema where nanomolar concentrations of E2 can decrease the firing rate most probably by increasing BK current^35^. All these examples underscore the physiological importance of the regulation of BK channels by E2 and made worthwhile efforts in determining the molecular nature of the interaction between this hormone and the BK channel.

Here, we expressed the β1 subunit alone or co-expressed with the BKα subunit, and demonstrated that E2 binding did not require the presence of the BKα subunit. We established that E2 increases *P*_*o*_ by stabilizing the voltage sensor in its active configuration and increasing the coupling between voltage sensor activation and pore opening. Notably, we found that E2 binds to a hydrophobic cluster of residues, E2-binding pocket, in the second transmembrane segment of β1. Furthermore, we showed that residues tryptophan 163 and phenylalanine 166 in β1 subunit, are necessary to stabilize E2 in the E2-binding pocket, since substitution of these residues eliminated the E2 effect on BKα/β1 channel.

## Results

### E2 binds to the β1 subunit

**Figure 1:**
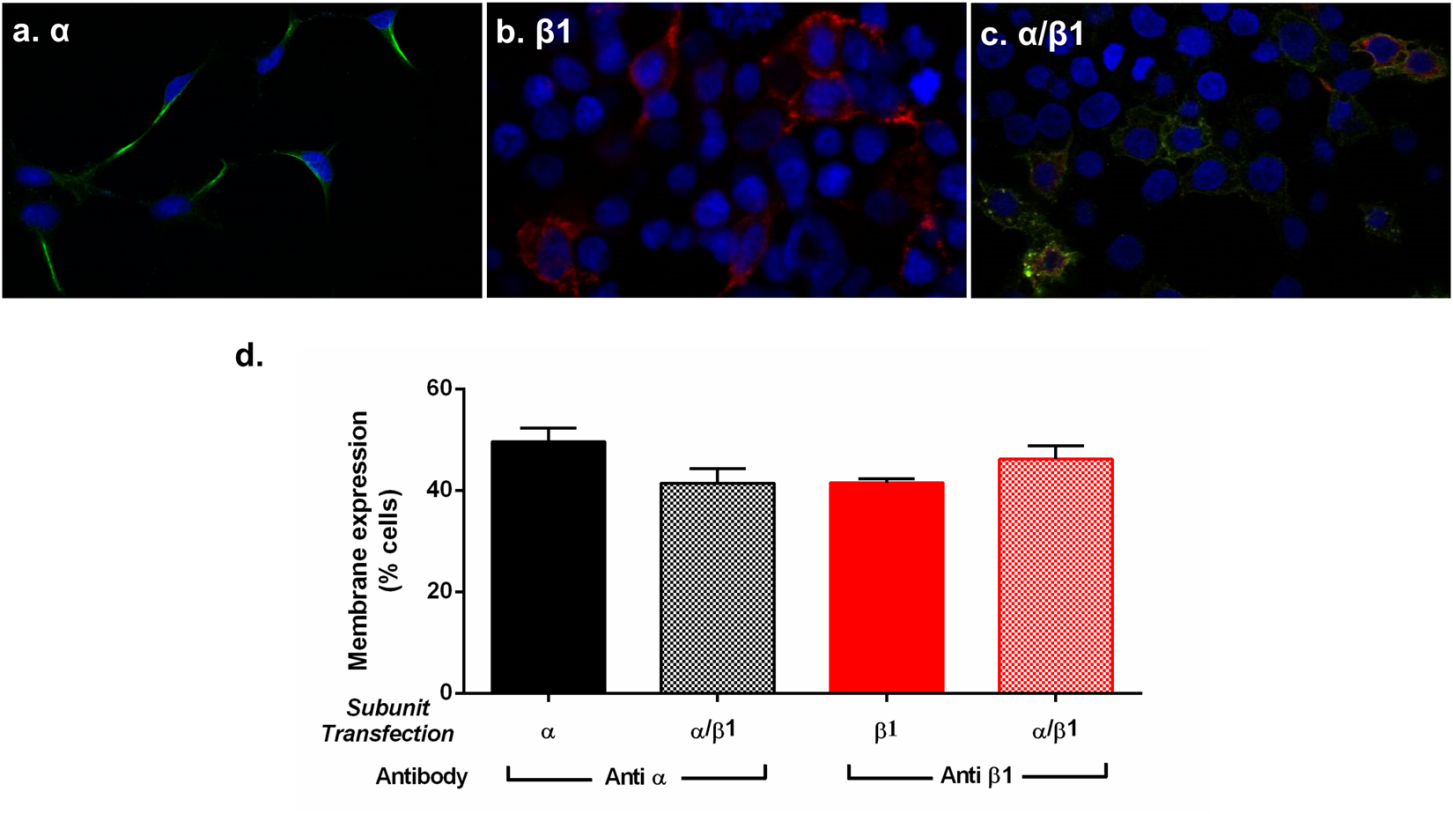
BK channel expression in HEK 293 cells. **(a)** Immunofluorescence of channels formed by the α subunit; green: anti-KCa1.1 and Alexa Fluor 488 antibodies. **(b)** Immunofluorescence of the β1 subunit; red: anti-MaxiK- β1 and Alexa Fluor 568 antibodies. **(c)** Immunofluorescence of channels formed by co-expression of α/β1 subunits; yellow: merged image of green and red antibodies in cells expressing α and β1 subunits. Blue: DAPI nuclear stain. **(d)** Quantification of membrane expression by flow cytometry (% cells). α: 49.2 ± 2.7; β1: 41.51 ± 0.8, α in α/β1 co-transfected cells: 41.4 ± 2.8; β1 in α/β1 co-transfected cells: 46.1 ± 2.6. Error bars: standard error of mean (SEM).

We studied the E2.binding in BK channels by transfecting HEK 293 cells with BKα, β1 and BKα/β1 subunit, respectively. Membrane expression was evaluated by immunocytochemistry and flow cytometry (Fig. 1). The α and β1 subunit reach the plasma membrane when expressed alone or as a α/β1 complex (Fig. 1a–c). Membrane expression was quantified using extracellular labeling and flow cytometry and was detected in 30–40% of the studied cells (Fig. 1d).

**Figure 2:**
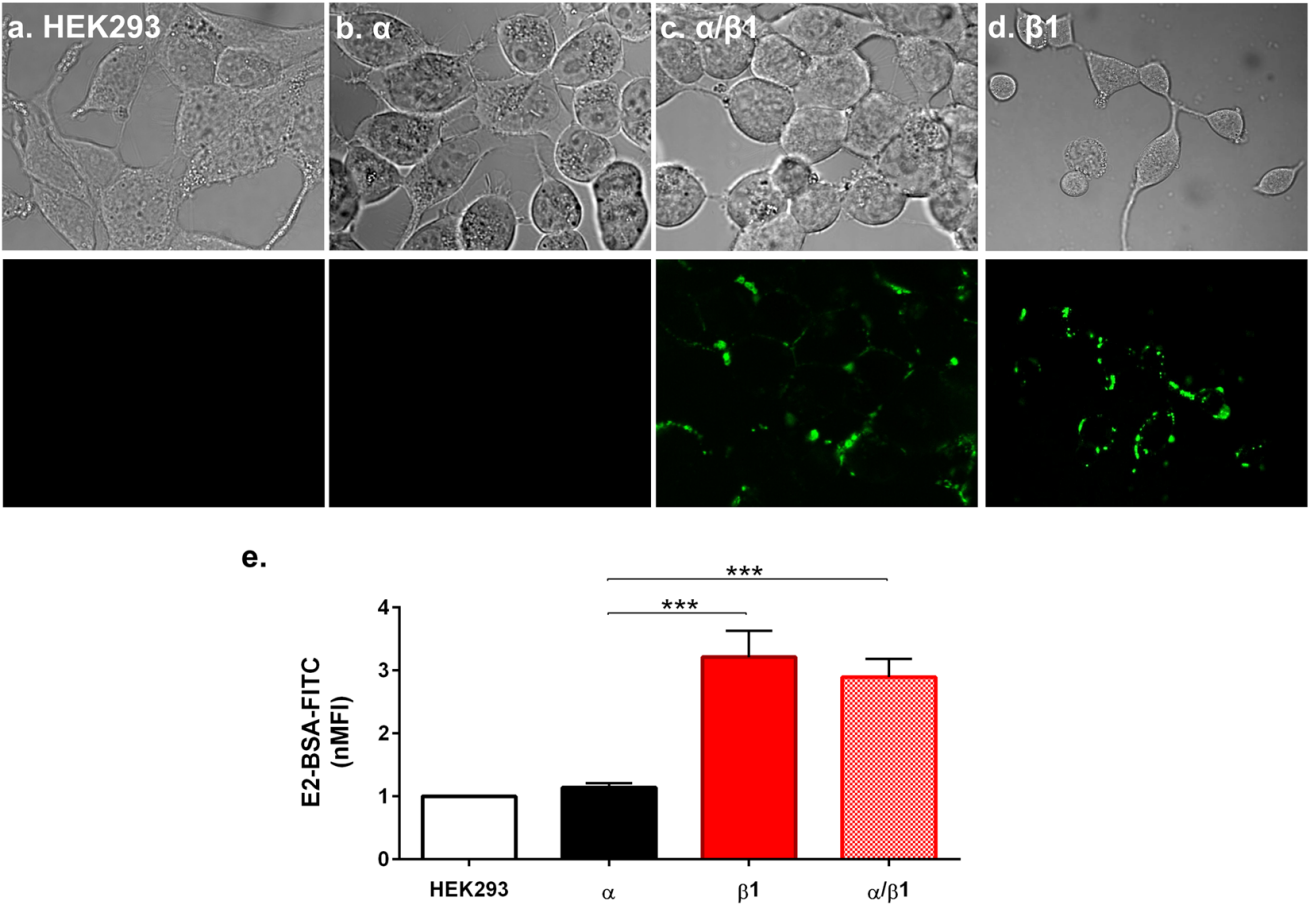
E2 binding to BK channel. **(a)** Representative images of HEK 293 cells without transfection, **(b)** transfected with α subunit, **(c)** cells transfected with α/β1 subunits, and **(d)** cells transfected with β1 subunit, showing bright field and FITC channels. All cells were treated with 10 μM E2-BSA-FITC. **(e)** Membrane binding quantification of E2-BSA-FITC by flow cytometry using a normalized Median Fluorescence Intensity (nMFI). α: 1.14 ± 0.07 (black bar), β1: 3.21 ± 0.43 (red bar), α/β1: 2.89 ± 0.29 (squared pattern red bar). Error bars: SEM, ***P < 0.001.

We explored the binding characteristics of E2 to the BK channel by E2-binding assays using a membrane-impermeant conjugate of fluorescein isothiocyanate-labelled E2 covalently linked to albumin (E2-BSA-FITC) as described in Methods. The binding was analyzed by confocal microscopy and quantified by flow cytometry (Fig. 2). In support of our previous results^19^, E2-BSA-FITC binds to α/β1 expressing cells (Fig. 2c,e) with an normalized Median Fluorescence Intensity (nMFI) of 2.89 ± 0.29 but not to control untransfected cells or to BKα expressing cells (Fig. 2a,b,e). Also, E2 binds to cells expressing the β1 subunit alone, with an nMFI of 3.21 ± 0.43, suggesting that the E2-binding site is located in the β1 subunit (Fig. 2d,e).

### E2 stabilizes the voltage sensor in its active configuration

**Figure 3:**
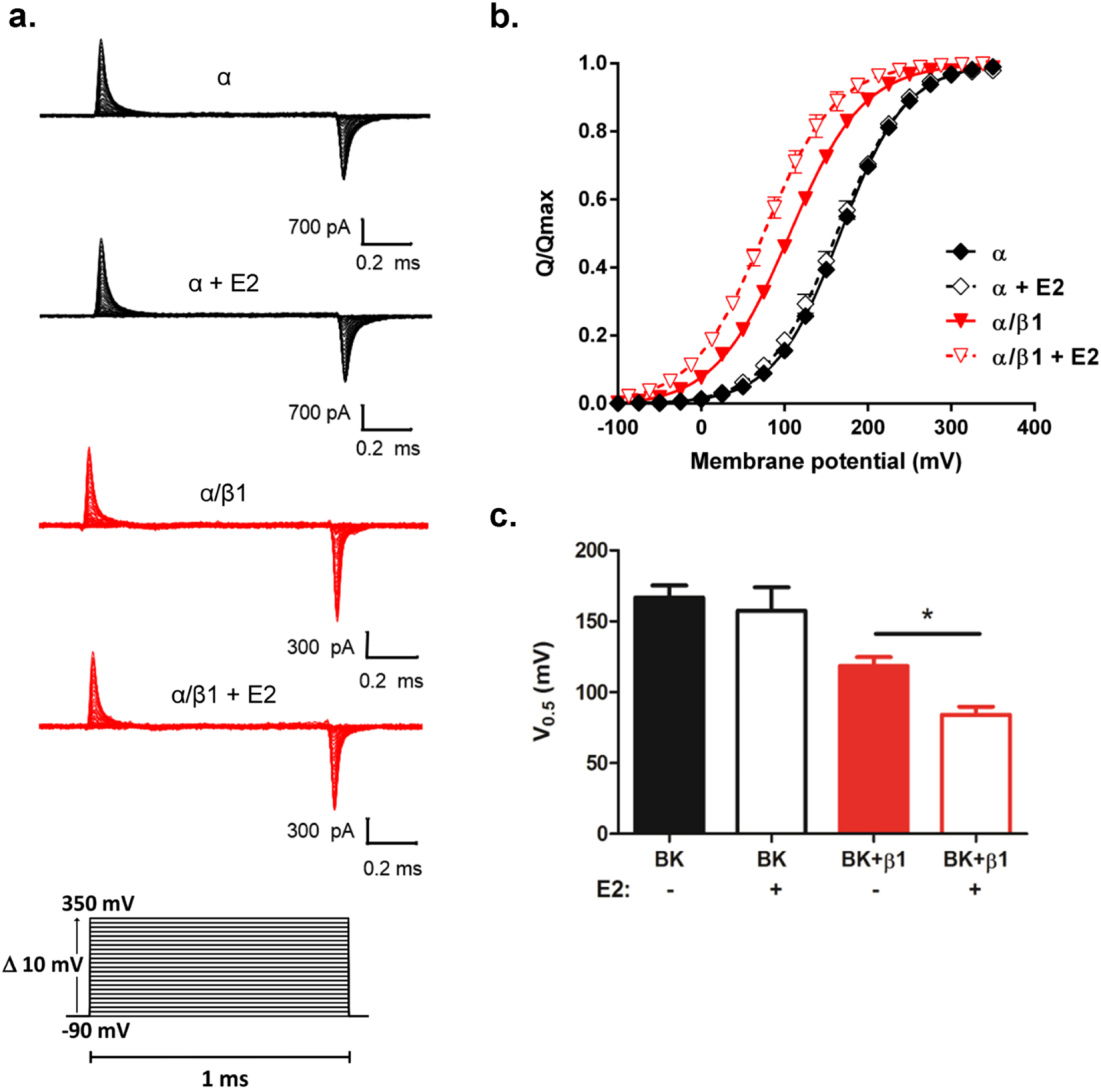
E2 modulates VSD equilibrium in BKα/β1 channels. **(a)** Representative gating current recordings from α channels (black traces), and α/β1 channels (red traces) in absence or presence of E2, as indicated. **(b)** Gating charge-voltage relationships for α (◆) and α/β1 channel complexes (). Q-V relationships for α exposed to E2 (♢) and α/β1 subjected to E2 (), are also shown. **(c)** Quantification of V_0.5_ obtained from the Q-V curves from α channels (black bars) and α/β1 channels (red bars), with or without E2 as indicated (mean ± SEM). Non-parametric *t*-tests were used to compare results between channels and experimental conditions. *P < 0.05.

E2 can act as an activator of the BK channel even in the absence of internal Ca^2+ ^^19^. However, its mechanism of action is unknown. β1 modulates BK channel activity by displacing VSD equilibrium towards the active state^14,16^. In order to examine the possible effects of E2 on resting-active VSD equilibrium, we measure gating currents. Gating currents reflect the displacement of the charges contained in the voltage sensor. We measured gating currents to examine the effect of E2 on BK modulation by β1 in *Xenopus laevis* oocytes expressing BKα and BKα/β1 channel (Fig. 3). We were able to detect robust gating currents in the absence or in the presence of E2 (Fig. 3a). E2 shifted the Q-V curve 35 mV to the left along the voltage axis in channels composed of α/β1 subunit (Fig. 3b,c), but did not affect the gating currents of channels formed by the α subunit alone (Fig. 3b,c). These results suggest that E2 targets the β1 subunit modifying the resting-active voltage sensor equilibrium of BKα/β1 channel. The β1 subunit stabilizes the voltage sensor domain (VSD) of BK channels in its active configuration^14,16^. Notably, E2 strengthened this stabilization of the VSD in BKα/β1 channel, shifting the half voltage of the Q-V curve, *V*_0.5_ to the left, with no change in the Q/V curve slope suggesting that the equivalent charge per voltage sensor is unaltered by the hormone (Fig. 3c).

### E2 binding site is located in a hydrophobic pocket in TM2 of the β1 subunit

**Figure 4:**
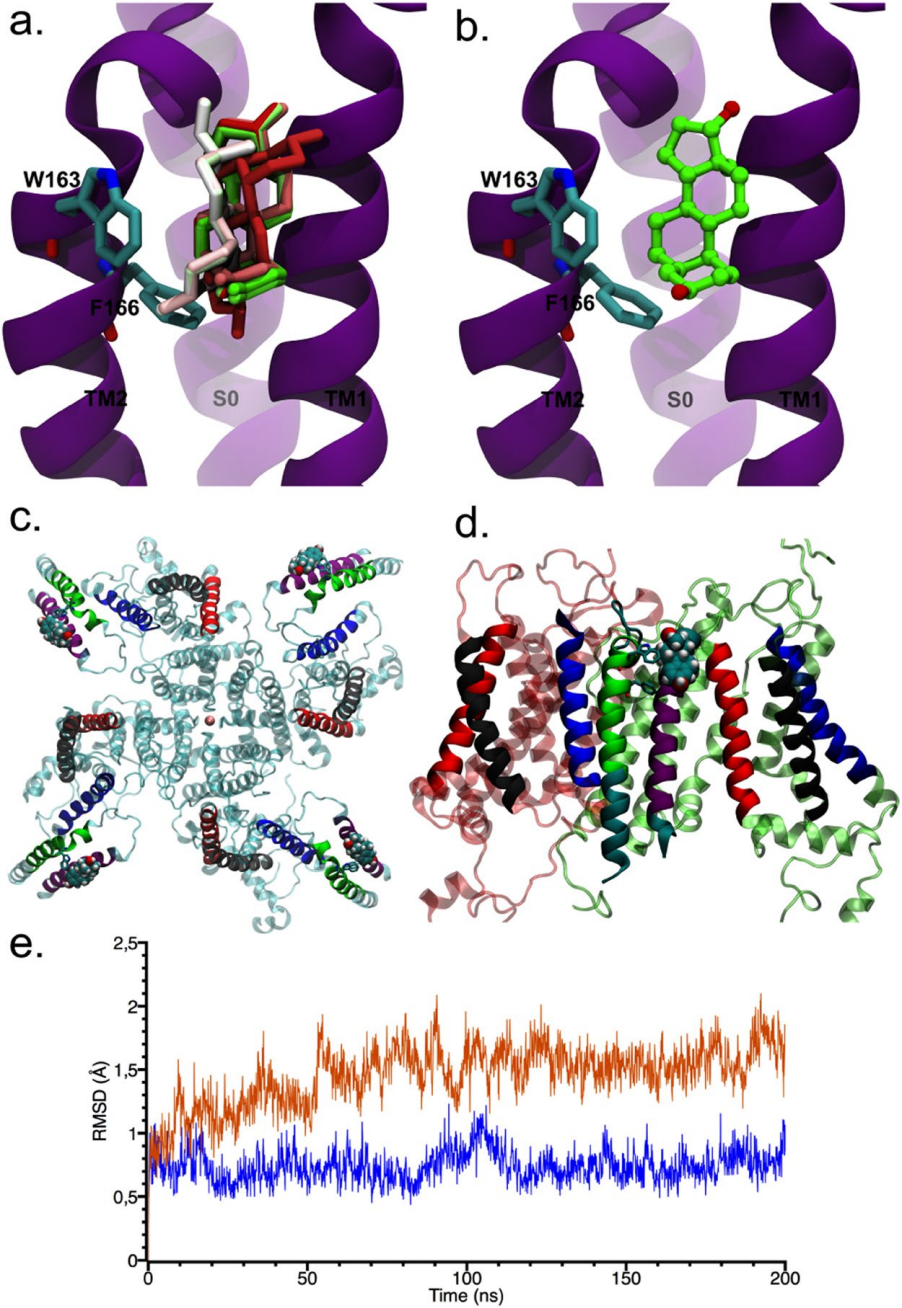
Molecular modelling of E2 interaction with BK β1 subunit. **(a)** Docking simulation of E2 into the β1 subunit. The sampling conformations were performed using the whole β1 and the E2 conformations with the better 10 docking scores are shown. **(b)** The E2 molecule with the best docking score (amino acid residues at less than 3 Ǻ from E2) is shown close to hydrophobic residues W163, and F166. **(c)** An upper view of the whole BK α/β1/E2 channel complex. Note that the β1 subunit resides between two adjacent voltage sensor domains. **(d)** Lateral view of the BK α/β1/E2 channel complex. Color code: Blue: S0; Red:S1; Grey:S2. For the sake of clarity, the β1 transmembrane segments were colored differently. TM1: violet; TM2:green. The E2 molecule is shown in CPK. **(e)** Backbone RMSD profile of β1 TM segments of the β1/E2 complex in a membrane system (orange), and RMSD profile of β1 TMs in the complex α/β1/E2 (blue). Last 200 ns of two trajectories are shown.

We further investigated the potential residues involved in E2 binding to the β1 subunit by computational modeling of the β1 subunit, a systematic structure-activity relationship modulation and a docking study (Fig. 4). E2-docking simulations to the β1 subunit showed the existence of a hydrophobic cluster of residues (W163 and F166) in the second transmembrane segment (TM2) of β1 that can define an E2 binding pocket and that E2 is intercalated between the β1 subunit transmembrane segments (Fig. 4a,b). These results hint that π−stacking and Van der Waals interactions between the tryptophan 163 (W163) and the benzyl ring of E2 are partially responsible for the E2 binding, which was further stabilized through additional hydrophobic interactions (Fig. 4b), in particular with the phenylalanine 166 (F166).

In an attempt to position the β1 subunit in the α/β1 complex, we used the S0 transmembrane domain of the α subunit as a reference for locating the β1 subunit in the complex. The molecular modeling of the complex show (Fig. 4c,d) that the β1 subunit resides between the voltage sensors of two adjacent α subunits. TM2 is in the neighborhood of S0 and that TM1 is close to the S1 and S2 segments. Notably, this result agrees with those of Liu *et al*.^36^, that by determining the extent of crosslinking between cysteines located in the β1 and the α subunit, concluded that the extracellular end of TM2 makes contact with S0 and that TM1 is near to both S1 and S2. Our modelling also gives a natural explanation as to why E2 binds only to β1 and not to α. A 400 ns molecular dynamics (MD) simulation of the two transmembrane segments of β1 in the β1/E2 complex inserted into a lipid bilayer surrounded by a 0.15 M KCl solution revealed that both β1 transmembrane segments remain parallel to each other during the last 200 ns of MD (Fig. 4e, orange). On the other hand, the last 200 ns MD simulation of complex α/β1/E2 inserted into a lipid bilayer surrounded by a 0.15 M KCl solution of a 400 ns MD, shows that the transmembrane segments of β1 in complex α/β1 is more stable than β1 membrane system (Fig. 4e, blue).The E2 interaction is mainly with residues W163 (99% of the time of simulations) and F166 (73%) in TM2 also the simulation of the α/β1/E2 complex reveals that E2 binds to the β1 surface that is in contact with the lipid bilayer as is observed in the Fig. 4c,d.

### Mutants W163I and F166A in TM2 abolish binding of E2 to β1

**Figure 5:**
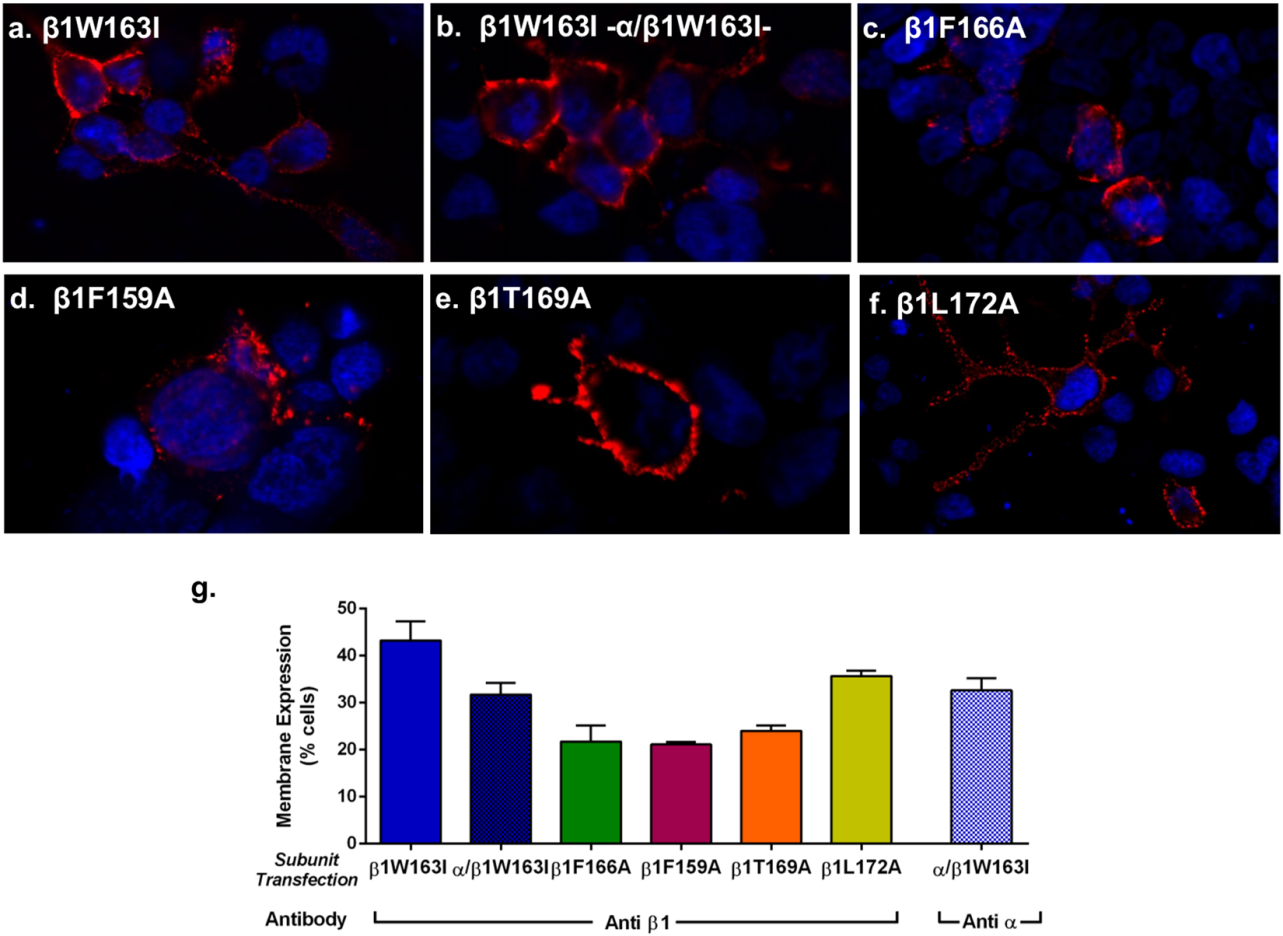
Mutant BK β1 channel expression in HEK 293 cells. **(a)** Immunofluorescence of HEK cells showing plasma membrane expression of β1 mutant W163I expressed alone, **(b)** or in combination with α subunit. **(c)** Immunofluorescence of β1 mutant F166A expressed alone, **(d)** β1 mutant F159A **(e)** β1 mutant T169A and **(f)** of β1 mutant L172A. **(g)** Quantification of levels of membrane expression measured by flow cytometry (% cells), β1W163I: 43.21 ± 4.1 (blue bar); β1, α/β1W163I: 31.71 ± 2.4 (blue and black squared pattern) using an antibody against β1 when α/β1W163I were co-transfected; β1F166A: 21.74 ± 1.9% (green bar); β1F159A: 21.10 ± 0.3% (purple bar); β1T169A: 23.98 ± 1.1 (orange bar); β1L172A: 35.63 ± 1.1 (yellow bar); antibody against α when α/β1W163I were co-transfected (blue and white squared pattern bar): 32.59 ± 2.5; Error bars: SEM. Red: anti-MaxiK-β1and Alexa Fluor 568 antibodies; blue: DAPI nuclear stain.

We explored the role of W163 as a possible site of interaction for E2 with the β1 subunit by substituting it with isoleucine (β1W163I). Isoleucine was used because it is a residue as hydrophobic as tryptophan but lacks the aromatic ring that putatively interacts with E2. As observed before for β1 subunit, the mutant β1W163I is efficiently detected in the plasma membrane when expressed alone (43.21 ± 4.08%) or in combination (31.71 ± 2.48%) with the α subunit (α/β1W163I) (Fig. 5a,b,g). We further explored the role of F166 in the binding of E2 due to its close location to the W163 residue and its possible association with the stabilization of the binding through hydrophobic interaction. We replaced the phenylalanine in position 166 by an alanine (β1F166A), transfected the mutant and investigated the expression in plasma membrane alone or in combination with the BKα subunit. The mutant β1F166A expressed well in the plasma membrane, and quantification using flow cytometry showed it does in 21.74 ± 1.9% of HEK cells (Fig. 5c,d).

We also used the F159 residue, located towards the N-terminus of TM2 but outside the hydrophobic pocket as a negative control for the binding experiments. We found that the β1F159A mutant expresses alone in the plasma membrane in a 21.10 ± 0.3% of the cell population (Fig. 5d,g). On the other hand, point mutations in TM2 of the β1 subunit (T169A and L172A) were previously shown to eliminate the effects of lithocholate, a steroid with a similar structure to E2 on the BK channel^37^. To determine if the binding sites for lithocholate and E2 were the same or overlapping, we also produced β1 subunits containing the mutations β1T169A and β1L172A. These β1 mutant subunits were expressed in the plasma membrane and quantification indicates that 22.3 ± 0.93% of cells expressed β1T169A and 35.63 ± 1.15% β1L172A (Fig. 5e–g).

**Figure 6:**
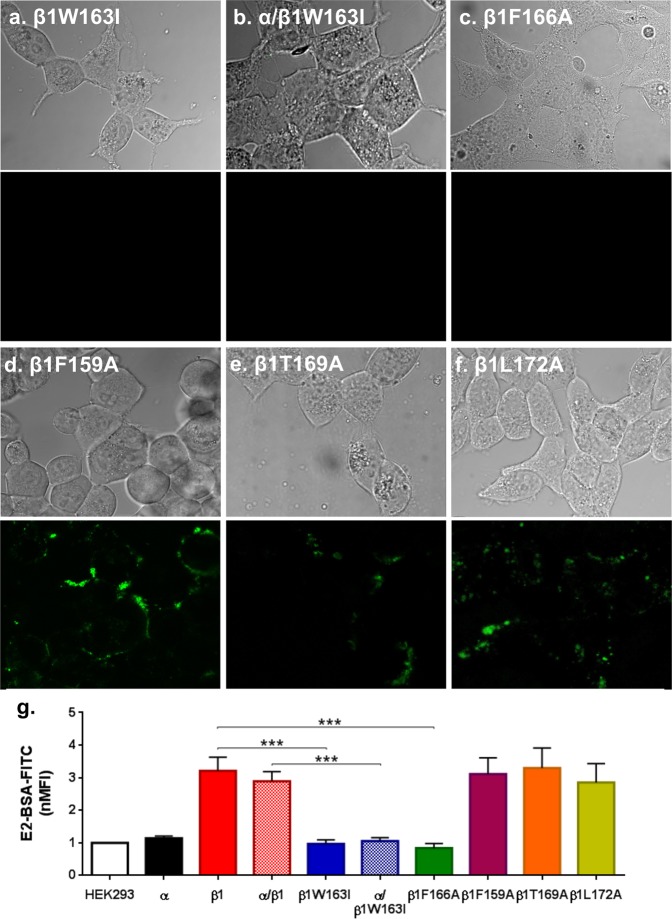
E2 binding to different β1 subunit mutants. **(a)** Cells transfected with β1 mutant β1W163I. **(b)** Cells transfected with α/β1W163I. **(c)** Cells transfected with β1F166A. **(d)** Cells transfected with β1 mutant β1F159A. **(e)** Cells transfected with β1 mutant β1T169A. **(f)** Cells transfected with β1 mutant β1L172A. Upper: bright-field; bottom: fluorescence field. **(g)** Membrane binding quantification of E2-BSA-FITC by flow cytometry in nMFI of β1W163I: 0.97 ± 0.1 (blue bar); α/β1W163I: 1.05 ± 0.1 (blue and white squared pattern bar); β1F166A: 0.84 ± 0.1 (green bar); β1F159A: 3.33 ± 0.1 (purple bar); β1T169A: 3.30 ± 0.6 (orange bar); β1L172A: 3.37 ± 0.6 (yellow bar). Error bars: SEM, ***P < 0.001.

E2 binding experiments showed that there is no steroid binding to cells transfected with β1W163I, α/β1W163I or β1F166A (Fig. 6a–c,h), after incubation with 10μM of E2-BSA-FITC. These results, together with the docking study, suggests that the W163 and F166 residues are crucial for the interaction between E2 and β1. We evaluated the roles of β1 mutants F159A, T169A and L172A in E2 binding to the β1 subunit. Binding assays demonstrated that these point mutations alone or in combination with the BKα subunit behaved similarly to the wild-type β1 subunit (Fig. 6d–g). Quantification using flow cytometry showed a nMFI of 3.33 ± 0.6 for β1F159A, 3.30 ± 0.6 for β1T169A and 2.85 ± 0.6 for β1L172A thus ruling out any involvement of these residues in the E2- binding site. This evidence leads us to conclude that even though the F159 residue has the same characteristics as the F166, it is not part of the binding site of E2. Also, even though there is evidence of their role of T169A and L172A in the binding of other steroids^37^, our results showed that they are not part of the E2-binding site.

### α/β1W163I and α/β1F166A BK channels are not activated by E2

**Figure 7:**
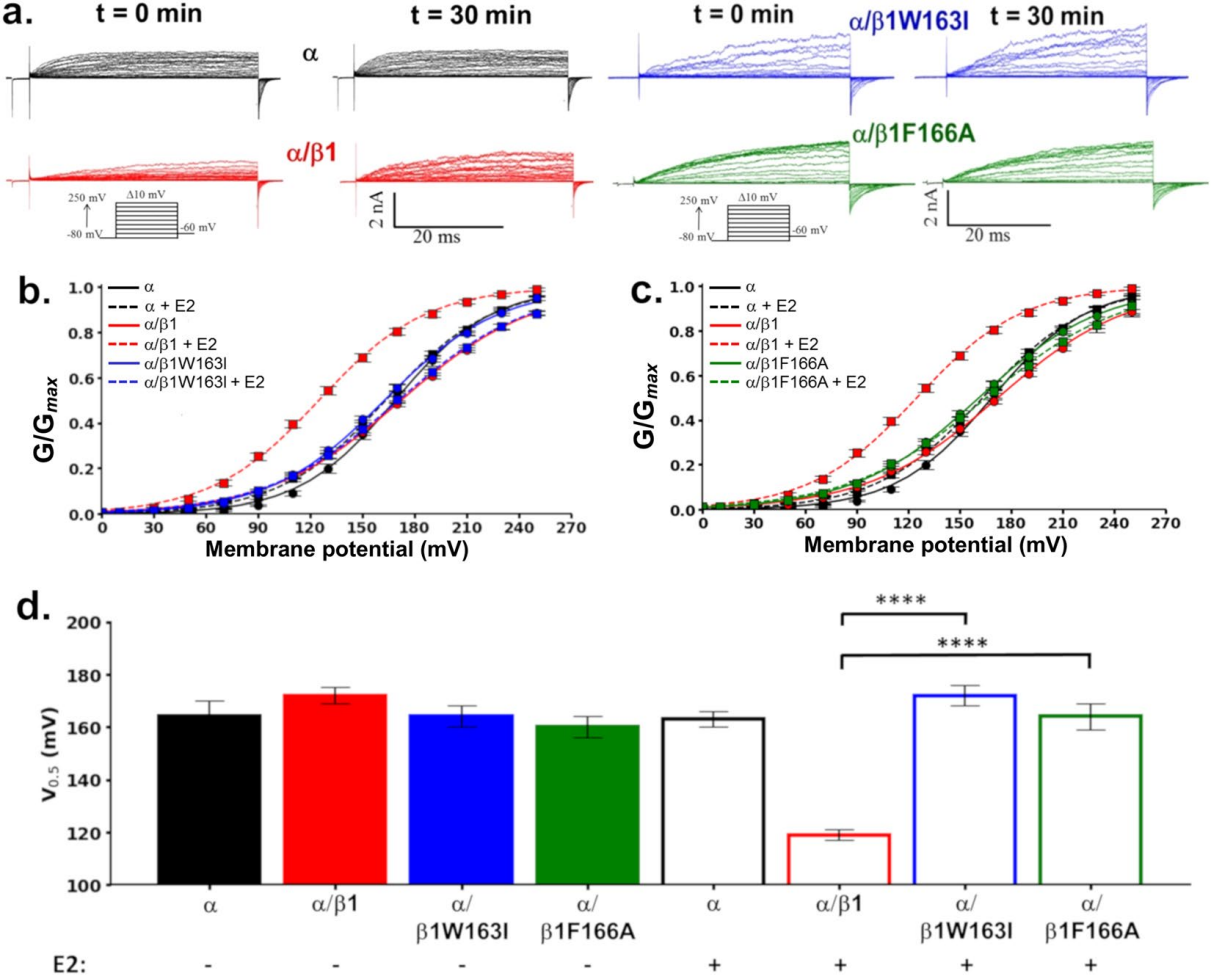
E2 effect on BK channel macroscopic currents. **(a)** Representative macroscopic ion current recordings from α channels (black traces), α/β1 channels (red traces), α/β1W163I (blue traces) and α/β1F166A (green traces) without 10 μM E2 at time 0 min or with E2 at 30 min. **(b**,**c)** Conductance-voltage relationships for α (black), α/β1 (red), α/β1W163I (blue) and α/β1F166A (green) channel complexes before (circles) and after exposition to 10 μM E2 (squares). Lines represent the best Boltzmann fit. **(d)** Quantification of V_0.5_ obtained from the G-V curves from BK α channels (black bars), α/β1 channels (red bars), α/β1W163I channels (blue bars) and α/β1F166A channels (green bars)with or without E2 as indicated (mean ± SEM). Differences between channels and experimental conditions were analysed using *t*-test. Half activation voltages (V_0.5_) were: α: 164 ± 6 mV (black filled bar); α/β1: 173 ± 3 mV (red filled bar); α/β1W163I: 164 ± 4 mV (blue filled bar), α/β1F166A: 160 ± 4 mV (green filled bar); α + E2: 163 ± 3 mV (black empty bar); α/β1 + E2: 119 ± 2 mV (red empty bar); α/β1W163I + E2: 172 ± 4 mV (blue empty bar), α/β1F166A + E2: 164 ± 5 mV (green empty bar). ****P < 0.0001. n = 5–8.

Wild type BK channels alone or in combination with the β1 subunit responded to Ca^2+^ and E2 as reported previously^19^ (Fig. 7a–d and Supplementary Fig. S1). We compared the activation kinetics when exposed to Ca^2+^ for BKα, α/β1, α/β1W163I and α/β1F166A channels. The results show that the β1W163I and β1F166A mutants do not alter BK channel gating properties when co-expressed with α subunit (Supplementary Fig. S1).

The addition of E2 does not change the electrophysiological properties of the BK channel when the BKα subunit is expressed alone (Fig. 7a–d). Confirming our previous results^19^, BKα/β1 channels robustly respond to E2 in the nominal absence of Ca^2+^(∼20 nM) by shifting the V_0.5_ of GV curve about 50 mV to the left along the voltage axis, indicating that E2 is able to modify BK channel activity only when co-expressed with β1 (Fig. 7b–d). As predicted by the binding assays, BK α/β1W163I and α/βF166A channels were insensitive to E2, with virtually the same phenotype as BKα expressed alone (Fig. 7a–d). This result was in line with the observation that these channels behaved similarly to BKα/β1 channels regarding their activation kinetics and Ca^2+^ and voltage sensitivities (Supplementary Figs S1 and S2). In contrast, E2 did not promote changes in the macroscopic currents in the case of the α, α/β1W163I channels and α/β1F166A, but in the case of α/β1 channels these currents peaked at 5 min after the addition of E2 and they remained constant for 30 min (Supplementary Fig. S3).

**Figure 8:**
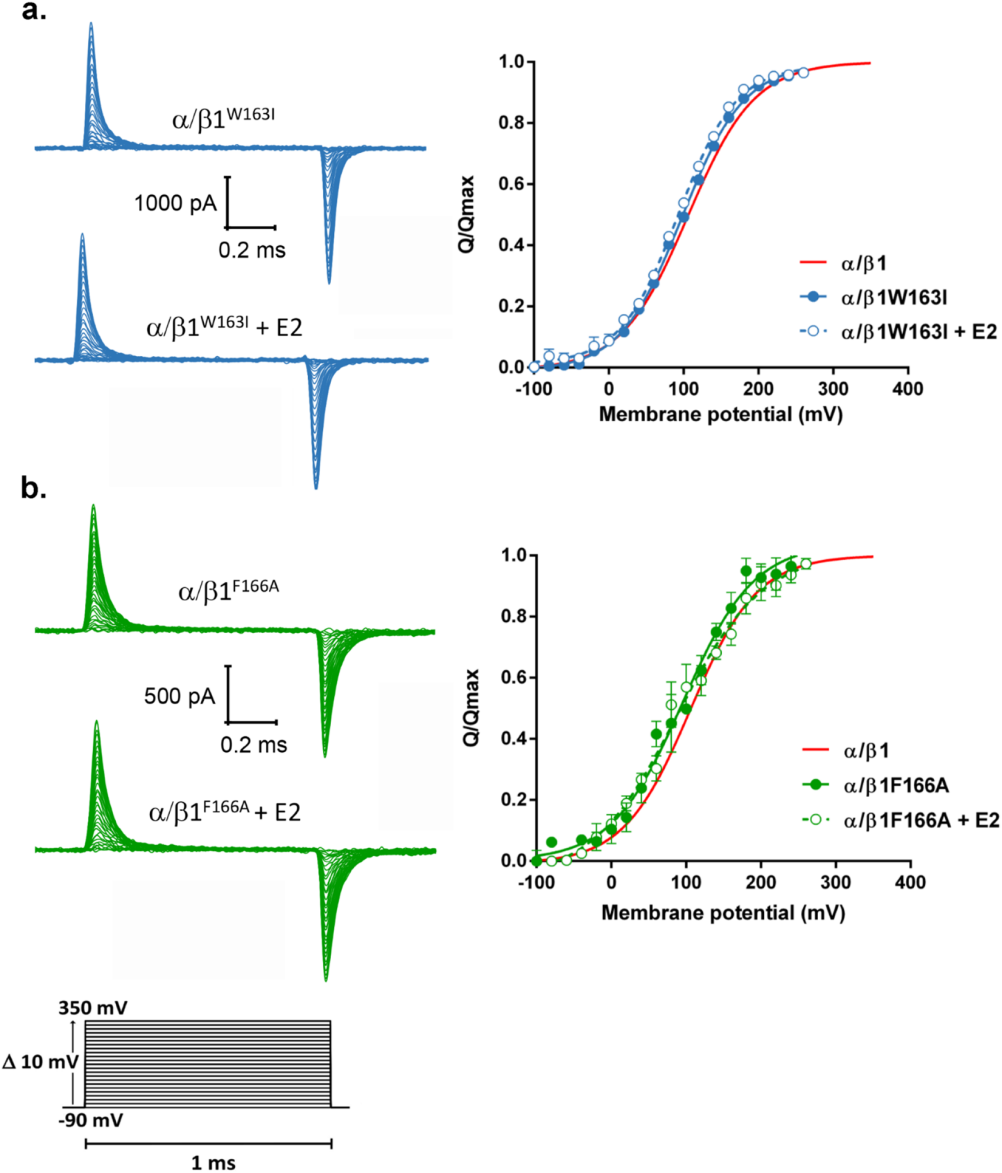
E2 is unable to modulate VSD equilibrium in α/β1W163I and α/β1F166A mutant channels. **(a)** Left: representative gating current recordings from α/β1W163I channels (blue traces), in the absence or presence of E2, as indicated. Right: Q/Qmax versus membrane potential relationships forα/β1W163I in control (filled blue circles) and after the addition of E2 to a final concentration of 10 μM (open blue circles). n = 3. **(b)** Left: representative gating current recordings from α/β1F166A channels (green traces), in the absence or presence of E2, as indicated. Right: Q/Qmax versus membrane potential curves for α/β1F166A in control (filled green circles) and after the addition of E2 to a final concentration of 10 μM (open green circles), n = 3. The red line corresponds to the Boltzmann function that best fit the α/β1 channel data.

Additionally, we investigate the effect of E2 on the VSD resting-active equilibrium in BK α/β1W163I and α/β1F166A channels, by measuring gating currents in *Xenopus laevis* oocytes (Fig. 8). We detected robust gating currents for both mutant channels in control conditions and the presence of E2. As shown in Fig. 8, mutations W163I and F166A in the β1 subunit maintain general features of the β1 wild type on the VSD equilibrium displacement, i.e., produce a stabilization of the VSD in its active configuration. However, in contrast to wild type BKα/β1 channels, in those BK channels formed by either W163I or F166A β1 mutant, E2 is ineffective to produce an additional effect on the VSD equilibrium. Our data strongly support that E2 binds to the β1 subunit, and that is specifically interacting with residues W163 and F166 residues in TM2.

## Discussion

BK channel expression is ubiquitous, but their function and cellular and tissue specificity are dependent on their co-assembly with auxiliary proteins, such as β1 subunits, which are responsible for ensuring the correct biological context of the channel’s activity^7,14^. In this study, we demonstrated that wild-type and mutant β1 subunits could be expressed in the plasma membrane without the BKα subunit, and that hydrophobic residues located in TM2 of β1 subunit are responsible for estradiol binding.

β subunits not only provide diversity in the physiological functions of the BK channel by modifying its dynamic range of response but have also been shown to be indispensable for mediating the modulation of BK channels by specific molecules, producing dramatic changes in their pharmacological properties^6^. Several steroid-like molecules such as dehydrosoyasaponin-I have been found to activate BK channels only when co-expressed with β1, increasing the open probability and causing an 80 mV leftward shift in V_0.5_^38–40^. A similar effect was observed with tamoxifen^28,31,41^. Other steroids, such as the adrenal androgen dehydroepiandrosterone, activated BK channels through the β2 subunit, while corticosterone shifted the voltage activation curve to the left when α and β4 subunits are co-expressed^18^. All these results suggest that BK channels require the β subunit to respond to steroids and that β subunits can differentiate between different types of steroids. We assessed the binding of E2 to the BK channel and demonstrate that E2 bound to a hydrophobic pocket at the TM2 of the β1 accessory subunit, highlighting the importance of this accessory subunit as a possible pharmacological target.

Litocholic acid is another steroid that has been shown to act as a BK channel modulator at micromolar concentrations (EC_50_ = 45 μM) only in when β1 is present^42,43^. Computational models and electrophysiological experiments led to the conclusion that residues T169 and L172 located in the TM2 of the β1 subunit^37^ formed part of the binding site for this estrogen analogue; however, the present study ruled out any involvement of these residues in the interaction between the E2 and the β1 subunit.

The β1 subunit increases the apparent BK channel Ca^2+^ sensitivity mainly by stabilizing the active configuration of the VSD but also it enhances Ca^2+^ binding to the RCK1 and Ca^2+^ bowl, with a large effect on channel gating^7,16,44^. Gating-current studies have shown that besides β1, β2 and β4 also stabilize the voltage sensors of the α subunits in their active conformation, while β3 does not alter the voltage sensor equilibrium^8,11,16,44–46^. The leftward shift of Q-V relationships induced by E2 entail a decrease in the energy needed to open the pore. E2 increases *P*_*o*_ by shifting the G-V curve to less positive voltages without affecting the channel voltage dependence. The question is then whether we can explain this result by an E2 effect on the resting-active equilibrium of voltage sensor (Fig. 3). We can explain this shift in Q-V with no change in the effective valence (Fig. 3) using a two-tiered allosteric model^47^. In this model the voltage-dependent equilibrium constant, *J*, that describes the resting (R)-active (A) equilibrium is given by Eq. (1):

1 $$J=\frac{A}{R}={J}_{0}{e}^{{z}_{J}FV/RT}={e}^{{z}_{J}F(V-{V}_{0})/RT}$$

where *J*_0_ is the equilibrium constant at 0 mV, *z*_*J*_ is the apparent number of gating charges per voltage sensor, *V* the membrane voltage, *V*_0_ the half-activation voltage of the Q-V curve, *R* is the universal gas constant, *T* is the absolute temperature (typically 295 K), and *F* is Faraday’s constant. Hence the leftward shift in the Q-V curve promoted by E2 with no change in *z*_*J*_ can be explained by an increase in *J*_0_. The ratio between the equilibrium constant after (*J*_0_*) and before (*J*_0_) E2 addition is thus given by Eq. (2):

2 $$\frac{{J}_{0}^{\ast }}{{J}_{0}}={e}^{\frac{{z}_{J}F({V}_{0}-{V}_{0}^{\ast })}{RT}}$$

Therefore, the change in *J*_*0*_ after the addition of E2 can be calculated from the change in Q-V half-activation voltage, and the new value can be introduced into the equation to calculate the open probability^47^, *P*_*0*_, in the absence of internal calcium (Eq. (3)):

3 $${P}_{O}=\frac{L{(1+JD)}^{4}}{L{(1+JD)}^{4}+{(1+J)}^{4}}$$

where *L* is the equilibrium constant that describes the closed-open reaction and *D* is an allosteric factor that couples voltage sensor activation with pore opening. Assuming that the values of *D* and *L* are as reported by Orio and Latorre^11^, the predicted change in half-activation voltage of the G-V induced by E2 is −15 mV. This value is less than the −53 mV change in V_0.5_ obtained experimentally (Fig. 7b,c), suggesting that E2 may modify other parameters of the model, related to channel activation. The full leftward shift of G-V promoted by E2 is recovered if the allosteric factor *D*, which couples voltage sensor activation with pore opening, is increased from 24^11^ to 30. Thus, the effects of E2 on BKα/β1 channel can be explained by an increase in the equilibrium constant that define the resting-active voltage sensor transition and in the coupling of voltage sensors with the pore.

We investigated the E2-binding site using an “*in silico”* approach with a molecular docking between the TM2 of the β1 subunit and E2, and identified a tryptophan residue at position 163 as an important constituent in this interaction, possibly due to a π-π interaction with the benzene ring of E2. π stacking interactions are a strong, noncovalent binding force that can form between the aromatic side chains of amino acids and other aromatic rings via electrostatic interactions^48,49^. These interactions are common between small molecules and proteins through the aromatic rings of amino acids such as tryptophan. Notably, alterations in these interactions have been correlated as basic chemical mechanisms in CVD, Alzheimer’s disease and cancer, thus increasing interest on them as potential pharmaceutical targets^50^. In the current study, we replaced tryptophan at 163 with an isoleucine residue, resulting in the loss of binding between E2 and the β1 subunit and abolishing the estrogen-activating effect on the BK channel. This interaction appears to be further stabilized by phenylalanine 166 residue through hydrophobic interactions. A similar effect has previously been demonstrated in human and rat α4β2 nicotinic receptors, where mutation of a single tryptophan in the C terminus of the α4 subunit eliminated the potentiation by E2, through a π-π interaction^51,52^. Furthermore, computational simulations of the effects on the crystal structure of Niemann Pick C2 Protein also revealed that π-π interactions stabilized the E2 binding through F66, V96 and T100 residues^53^.

The BKβ1 subunit has become a subject of interest because of its expression and involvement in the regulation of the cardiovascular tone. A gain-of-function polymorphism, with a single residue change (E65K) in the β1 subunit has been associated with increased Ca^2+^ sensitivity of the BK channel, resulting in more efficient feedback on vascular contractility, working as a protecting factor against myocardial infarction and stroke^54^. The above finding is of particular relevance in aging women, where the E65K mutation is a robust genetic factor associated with protection against CVD^13,54–56^. Hormone replacement therapy has also been an important subject of research, and loss of steroid hormones is known to increase the risk of CVD in women^25,57,58^. The direct relationships between E2 and both CVD and to BK channels highlights the importance of clarifying the role of this hormone in specific protein targets.

In summary, E2 binds directly to the β1 subunit of the BK channel through a hydrophobic pocket located in theTM2 mainly via the W163 and F166 residues. It also biased the resting-active equilibrium of the BK channel voltage sensor towards its active conformation and increased the coupling between the VSD and the pore opening. These results provide an explicit binding site and mechanism whereby E2 can modulate BK channel activity.

## Methods

### Reagents and antibodies

Membrane impermeable E2-BSA-FITC and soluble 17β-Estradiol (E2) were purchased from Sigma-Aldrich (St. Louis, MO, USA). Extracellular rabbit anti-MaxiK-α was purchased from Alomone Labs (Jerusalem, Israel), Intracellular goat anti-KCa1.1 antibody from Santa Cruz Biotechnology (Dallas, TX, USA) and extracellular rabbit anti-MaxiK-β1 from Novus Biologicals (Littleton, CO, USA). Nuclear marker 4′,6-Diamidino-2-Phenylindole, Dihydrochloride (DAPI) and secondary antibodies anti-rabbit Alexa Fluor 488, 568 and anti-goat Alexa Fluor 488 were acquired from Invitrogen (Waltham, MA, USA).

### Heterologous expression systems

#### Cell culture and gene transfection

HEK 293 cells were cultured in Dulbecco’s modified Eagle’s medium (DMEM), Gibco (Waltham, MA, USA) supplemented with 10% fetal bovine serum (FBS), Lonza (Basel, Switzerland) at 37 °C and 5% CO_2_. Once the cells reached 60% of confluence were transfected using Lipofectamine 2000 (Invitrogen) and human BKα (U11058) or BKβ1 (U25138) harbored in pcDNA3.1 plasmids kindly provided by L. Toro (University of California, Los Angeles, CA) with 1.5 μg of either the α, the β1 or a combination of both subunits in a 1:2 proportion of α:β1. Point mutations in the β1 subunit were made using a QuickChange kit from Agilent Technologies, (Santa Clara, CA, USA) following the manufacturer’s instructions. Cells used for the expression, binding assays and ionic current recordings were seeded on 12-mm glass coverslips coated with poly-D-lysine (Sigma-Aldrich).

#### Xenopus laevis oocytes

*X. laevis* oocytes were used to measure gating currents (Ig). mMESSAGE mMACHINE from Ambion (Waltham, MA, USA) was used for *in vitro* transcription of both subunits. The oocytes were injected in a proportion of 1:5 of α:β1 with 0.5 μg/μl of the respective RNA, 4–8 days before recording.

### Expression assays

For the immunochemistry assays, we used nonpermeabilized labeling for the membrane expression of α and β1 subunit and permeabilized labeling to investigate the co-expression of both. 24 hours after transfection, cells were seeded on 12-mm glass cover slide. We carried out nonpermeabilized labelling according to Bian *et al*.^59^. Briefly, live cells, were washed twice with PBS at 37 °C and incubated for 1 h at 4 °C with either MaxiK-α (1:250) or MaxiK-β1 (1:500) antibodies. The cells were washed twice with PBS and incubated with Alexa Fluor 488 conjugated anti-rabbit antibody (1:1000) for MaxiK-α and Alexa Fluor 568 conjugated anti-rabbit antibody (1:1000) for MaxiK-β1 for 1 h, followed by washing twice with PBS-FBS, and a fixing step with paraformaldehyde (3%), the nuclei were stained with DAPI (300 nM) for 15 minutes at room temperature, washed thrice in PBS, mounted with DAKO (Agilent Technologies, Santa Clara, CA, USA) and visualized with a confocal microscope. For permeabilized labelling, the cells were fixed with paraformaldehyde (4%) for 20 min, permeabilized with PBS and Triton X100 (PBS-T) (0.1%) with bovine serum albumin (2%) for 3 min. After two washes with PBS-T, the cells were incubated with intracellular goat anti-MaxiK-α (1:1000) and MaxiK-β1 (1:250) antibodies overnight at 4 °C, the secondary antibody and nuclei labelling, mounting and visualization were made as described above.

We quantified the BK channel membrane-expression using non-permeabilized labeling and flow cytometry. After a 24-hours transfection, live cells were washed twice with phosphate-buffered saline (PBS) at 37 °C, incubated for 1 h at 4 °C with either MaxiK-α (Alomone Labs, 1:50) or MaxiK-β1 (Novus, 1:100). Cells were then washed twice with PBS-FBS (2%) and incubated with Alexa Fluor 488 conjugated anti-rabbit antibody (1:300) for 40 min. After another two washes with PBS-FBS, α and β1 expression was verified in a BD Accuri™ C6 flow cytometer (BD Biosciences, San Jose, CA, USA).

### Binding assays

We carried out binding assays as described before^19^. To identify the E2 binding site we used confocal microcopy and quantified the binding with flow cytometry. For the confocal assays we used cells seeded on 12-mm glass cover slide and 24 hours after transfection, were incubated for 30 minutes with E2-BSA-FITC (10 μM) at 37 °C and 5% CO_2_ with DMEM and FBS 10%, washed twice with PBS at 37°and then visualized with a confocal microscope. For the binding quantification assays we used flow cytometry, suspended cells were incubated for 30 min with 10 μM of E2-BSA-FITC at 37 °C and with washed twice with PBS-FBS at 2% and measured with a BD Accuri™ C6 flow cytometer.

### Image acquisition and analysis

Cells were observed and images were collected by confocal microscopy using an Eclipse 80i Nikon (Nikon, Tokyo, Japan) with an 408 nm violet, 488 nm-argon and 543 nm-He-Ne laser diode lines, with a 450/35, 515/30 and 605/75 emission filters. Z-stack parameters were as follows: z-axis slices at ~1.0 μm intervals with a final Z-stack thickness of ~15 μm. Images were obtained using a water immersion 60X objective and the EZ-C1 Nikon program. Images were analyzed using ImageJ software (https://imagej.nih.gov/ij/ freely available from National Institutes of Health). The conditions, exposures and number of sections were identical for a specific experiment.

### Flow cytometry acquisition and analysis

For the flow cytometry quantification assays, each experiment consisted of three technical replicates and was repeated at least thrice. The run limit of the samples was set to 20000 gated events with a flow set to 22 μl/min events per sample. Samples were manually re-suspended before each acquisition and the cells were gated based on the light-scattering properties in the Side and Forward-scatter parameters, HEK293 cells without transfection were used as control and fluorescence was detected with the FL1 (530/533 filter) detector using the BD Accuri™ C6 software. All analysis were made using FlowJo® (Tree Star, Inc., Ashland, OR, USA) and normalizing as reported before. Briefly, we used the Median Fluorescence Intensity (MFI) of each sample and normalized it to the MFI of the HEK293 cells without transfection as a negative control using Eq. (4) ^60^.

4 $${nMFI}=\frac{{MFI}\,{sample}}{{MFI}\,{control}}$$

To compare among samples, the Shapiro–Wilk test and the Kruskal–Wallis non-parametric test (P < 0.01) was performed using GraphPad Prism 6 (GraphPad Software, Inc., CA, USA).

### Electrophysiology and data analysis

#### Ionic currents

Patch clamp recordings were carried out in transfected HEK293 cells on 12-mm glass coverslips coated with poly-D-lysine. 24 h after transfection, ion currents were recorded using the inside-out configuration to determinate the Ca^2+^-sensitivity of the channel and the E2 modulatory effect. Symmetrical solutions contained 140 mM KOH, 10 mM HEPES, 2 mM KCl, and a free Ca^2+^ concentration of ∼20 nM or 3 μM. All solutions were adjusted to pH 7.4 using methanesulfonic acid. Ca^+2^ was buffered with 5 mM EGTA (ethylene glycol-bis (β-aminoethyl ether)-N,N,N′,N′-tetraacetic acid), or NTA (nitrilotriacetic acid), according to the Ca^+2^ requirements. The Ca^+2^ concentrations were estimated using WinMaxChelator Software (http://www.stanford.edu/~cpatton/ maxc.html) and tested with a Ca^2+^ electrode (Thermo Scientific).

E2 experiments were conducted using the inside-out configuration; recordings were made in a ∼20 nM Ca^+2^ solution, to which 10 μM E2 solution was then perfused. Currents were elicited by 120 ms pulses at increasing voltages from −80 to 250 mV in 10 mV increments, followed by a step at −60 mV to measure tail currents. Measurements were made before the solution change and from the time of 5 min after, until 30 minutes. The maximum effect of E2 occurred after 5 min of exposure to E2. During recording, the E2 and different Ca^+2^ concentration solutions were applied to the membrane patches with a perfusion system. Borosilicate glass patch pipettes (1B150F-4, World Precision Instruments, Sarasota, FL, USA) with resistances of 2–4 MΩ were pulled in a horizontal pipette puller (Sutter Instruments, Novato, CA, USA) and fire-polished with a microforge (Narishige, Tokyo, Japan). All experiments were performed at room temperature (22–24 °C) using an EPC7 patch-clamp amplifier (HEKA, Lambrecht/Pfalz, Germany). The acquisition software was developed by Dr Patricio Orio using the LabView programming environment (National Instruments, Austin, TX, USA)^11^.

#### Gating currents

All Ig recordings were made in the inside-out patch-clamp configuration. The internal solution comprised 110 mM N-methyl-d-glucamine-MeSO_3_, 10 mM HEPES, and 5 mM EGTA. The external (pipette) solution contained 110 mM tetraethylammonium-MeSO_3_, 10 mM HEPES, and 2 mM MgCl_2_. The pH was adjusted to 7 with methanesulfonic acid. The internal Ca^2+^ concentration was assumed to be 0 in 5 mM EGTA.

Gating currents were elicited by 1-ms pulses at increasing voltages from −90 to 350 mV in 10 mV increments, as previously reported^61,62^. Ig recordings were made in excised patches containing α and α/β1 BK channels. Channels were exposed to E2 by perfusing excised patches with an internal solution with 10 μM E2, at least 5–10 times the volume of the chamber. After solution exchange, the maximum effect of E2 on Ig occurred after 5 min of exposure of the excised patch to the drug. Experiments were performed at room temperature (20–22 °C). Ig recordings in macro patches were carried out using borosilicate capillary glass pipettes as mentioned before. The pipette resistance was 400–900 kΩ (20–60 μm). Data were acquired with an Axopatch 200B amplifier (Axon Instruments, San Jose, CA, USA) and Clampex 10 acquisition software (Axon Instruments). Both the voltage command and current output were filtered at 20 kHz with an 8-pole Bessel low-pass filter (Frequency Devices). Current signals were sampled with a 16-bit A/D converter (Digidata 1322 A; Axon Instruments), using a sampling rate of 500 kHz. Leak subtraction was performed based on a P/4 protocol^62^.

#### Data analysis

All data analyses were performed with Clampfit 10 (Axon Instruments), Analysis software (kindly provided by Dr. Francisco Bezanilla), and Excel 2013 (Microsoft, Redmont, WA, USA). Gating currents were integrated between 0 and 400 μs after the voltage step to obtain the net charge movement. Q-V relationships were fitted with a Boltzmann function: Q = Q_max_/( 1 − exp(−zF(V − V_0_)/RT)), where Q_max_ is the maximum charge, z is the voltage dependency of activation, V_0_ is the half-activation voltage, T is the absolute temperature (typically 295 K), F is the Faraday’s constant and R is the universal gas constant. Q_max_, V_0_, and z were determined by using the solver complement of Microsoft Excel. Data were aligned by shifting them along the voltage axis by the mean ΔV_0 _ = (<V_0_> − V_0_), then binning them in a range of 25 mV, between −100 mV and up to 350 mV.

For the ionic currents, G-V relationships were fitted using a Boltzmann function: G = G_max_/(1 − exp(−zF(V −V_0,5_)/RT)), where G_max_ is the maximum tail conductance, z is the voltage dependency of activation and V_0.5_ is the half-activation voltage. G_max_, V_h_, and z were determined by using the solver complement of Microsoft Excel. Data were aligned by shifting them along the voltage axis by the mean ΔV_0.5_ = (<V_0.5_> − V_0.5_), then binning them in a range of 10 mV, between −80 mV and up to 250 mV. Data was analyzed with a non-parametric t-test (P < 0.05) using GraphPad Prism 6.

### Molecular model of β1-subunit and molecular docking of estradiol

A homology model of the α subunit of BK channel, sequence code Q12791 (UniProt), was built using the program Modeller^63^, as a reference we used the *Aplysia* BK channel structure (PDB: 5TJI) for transmembrane segments and the human BK channel (PDB: 3MT5) as template for the x-ray structure of intracellular domain (see alignment in Supplementary Information). Since the transmembrane loop of BK is longer than that of *Aplysia*, we performed a conformational sampling of this region using PSIPRED web service^64^. The structural model of the human BK channel was inserted in a POPC membrane of 200 × 200 Å. A water (TIP3) box was added and neutralized with a concentration of 0.15 mol/L of KCl. The system was submitted to energy minimization and subsequent equilibration protocols by 200 ns using AMBER 18 software^65^, and under NPT ensemble at 310.

The sequence code Q16558 (UniProt) of the β1 subunit of the BK channel was used to predict its molecular structure. The modelled sequence was reduced to the two β1-subunit transmembrane regions. Residues from 3–52 of the β1 primary structure define TM1 and TM2 is defined by residues 142–191. The external linker loop between the α helices was reduced to 10 amino acids. Given the lack of reference structures for the β1 subunit, an *Ab-initio* model was done using the QUARK online server^66,67^ to generate a 3D structure of the β1 subunit (see PDB in Supplementary Information). QUARK has been reported in CASP 2018^68^ as one of the best algorithms for ab-initio prediction. Its algorithm is based on a fragment-assembly method developed by the Yang Zhang Lab^66^. The structural fragments used by QUARK range from 1 to 20 residues, which are ensembled trough replica-exchange Monte Carlo simulation guided by an atomic-level knowledge-based force field. The two transmembrane segments were embedded into a phosphatidyloleoyl phosphatidylcholine (POPC) bilayer. Water molecules type TIP3P and 150 mM KCl complete the system. The whole system was then minimized and equilibrated for 300 ns. Electrostatic interactions were computed using particle mesh Ewald under periodic boundary conditions. The *Ab-initio* model of the β1-subunit was ensembled with the S0 segment of BK channel model structure. The minimum energy configuration of the β1-subunit and S0 segment complex was sampled by 50,000 configurations using a Monte Carlo calculation^69^. The different configurations were ranked according to the energy interaction between the β1 subunit and S0 segment of BK. The S0 segment was used as a reference to ensemble the complex β1 subunit-α subunit.

The molecular structure of E2 molecule was obtained of PubChem (PubChem code: DB00783) and prepared using LigPrep of Schrödinger 2019^70^ The force field applied to E2 was OPLS3^71^ The ionization state was defined at pH 7.0 +/− 2.0 using Epik software^72^. Stereoisomers and chiralities were determined directly from three-dimensional structure. The grid size used was 20 × 20 Å. The molecular docking of E2 was performed into a wild-type systems using the extra precision method, ligand-flexible sampling and adding Epik states penalties. All molecular docking simulations were performed using Glide 2019 from Schrödinger suite^73^. Ten configurations for E2 were chosen to do a post-docking minimization using a threshold of 0.5 kcal/mol.

Finally, the complex of the α/β1/E2 system (under the same conditions of solvation and neutralization of α subunit) was submitted to energy minimization and subsequent equilibrations protocol. Then, it was run 400 ns using AMBER 18 software, under NPT ensemble at 310. All the figures were done of this article were done using VMD software^74^.

## Supplementary information

Supplementary Information

Dataset 1

Dataset 2

## Data Availability

The data supporting the findings of this study are available upon request from the corresponding author (including data presented in the main text and in the Supplementary Information).
