# Supplementary material for: The molecular nature of the 17β-Estradiol binding site in the voltage- and Ca2+-activated K+ (BK) channel β1 subunit
Source: Sci Rep. 2019 Jul 10;9:9965. doi: 10.1038/s41598-019-45942-1 (PMC6620312; doi:10.1038/s41598-019-45942-1)
Supplement: Supplementary file 1 — Supplementary Information [file 41598_2019_45942_MOESM1_ESM.docx]

**The molecular nature of the 17β-Estradiol binding site in the voltage- and Ca^2+^-activated K^+^ (BK) channel β1 subunit**

Sara T. Granados^1,2^, Karen Castillo^2^, Felipe Bravo-Moraga^2,3^, Romina V. Sepúlveda^2,3^, Willy Carrasquel-Ursulaez^2^, Maximiliano Rojas^,3^, Emerson Carmona^2^, Yenisleidy Lorenzo-Ceballos^2^, Fernando González-Nilo^2,3^, Carlos González^2^, Ramón Latorre^2,*^, Yolima P. Torres^1,*^.

^1^ Departamento de Nutrición y Bioquímica, Facultad de Ciencias, Pontificia Universidad Javeriana, Bogotá, Colombia.

^2^ Centro Interdisciplinario de Neurociencia de Valparaíso, Facultad de Ciencias, Universidad de Valparaíso, Valparaíso, Chile.

^3^ Center for Bioinformatics and Integrative Biology, Facultad de Ciencias de la Vida, Universidad Andrés Bello, Chile.

**Supplementary Information**

**Supplementary Figure 1**

**
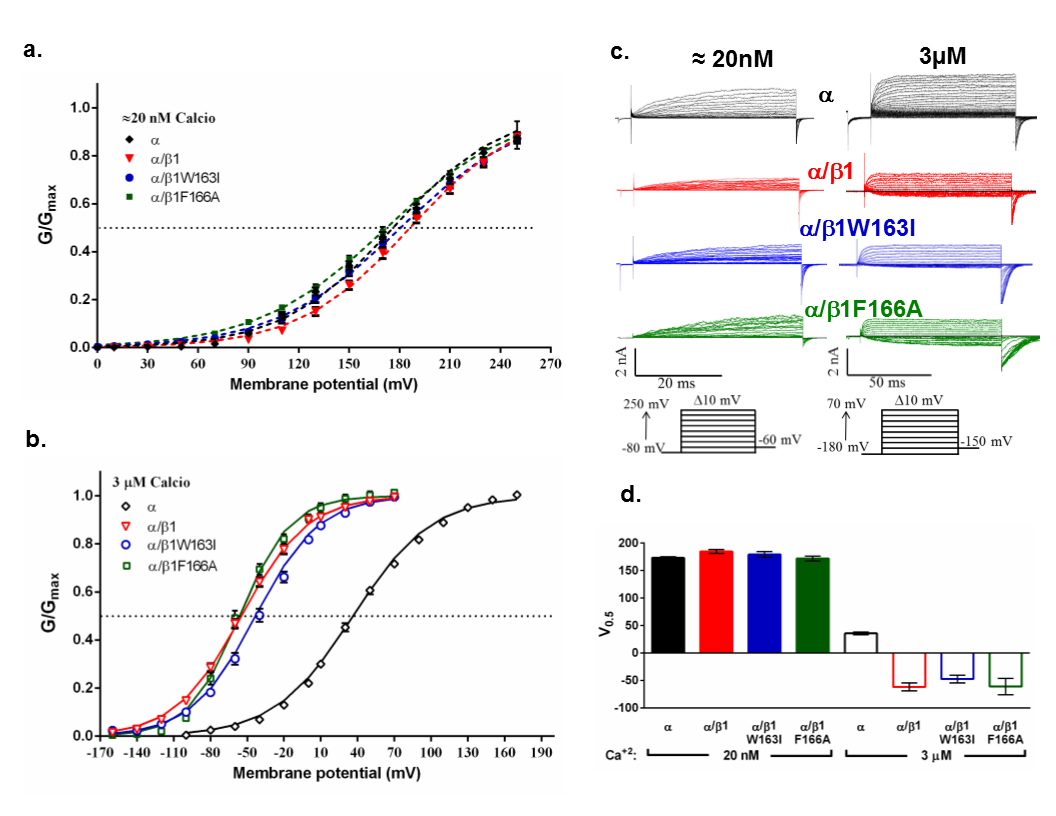
**

**Supplementary Figure S1. Effect of Ca^2+^ on macroscopic BKα/β1W163I and BKα/β1F166A currents.** **(a)** G/V curves for BKα channels (⬥), α/β1 channels (▼),α/β1W163I channels (●), and α/β1F166A channels (◼)at ~ 20 nM Ca^2+^.**(b)** G/V curves for BKα channels (**◇**), α/β1 channels (**▽**),α/β1W163I channels (**○**), and α/β1F166A channels (**🞏**) at 3 μM Ca^2+^, lines represent the best Boltzmann fit. **(c)** Representative current traces for ~ 20 nM and 3 μM Ca^2+^ **(d)** V_0.5_ at ~ 20 nM and 3 μM Ca^2+^. V_0.5_ at ~ 20 nM; α: 172±2 mV, n=5; α/β1: 185±4 mV, n=12; α/β1W163I: 179±5 mV, n=6, α/β1F166A: 172±4 mV, n=8 ; V_0.5_ at 3 μM α: 36±3 mV, n= 5; α/β1: −61±7 mV, n=5; α/β1W163I: −47±7 mV, n=5, α/β1F166A: -60±14 mV, n=5.

**Supplementary Figure 2**

**
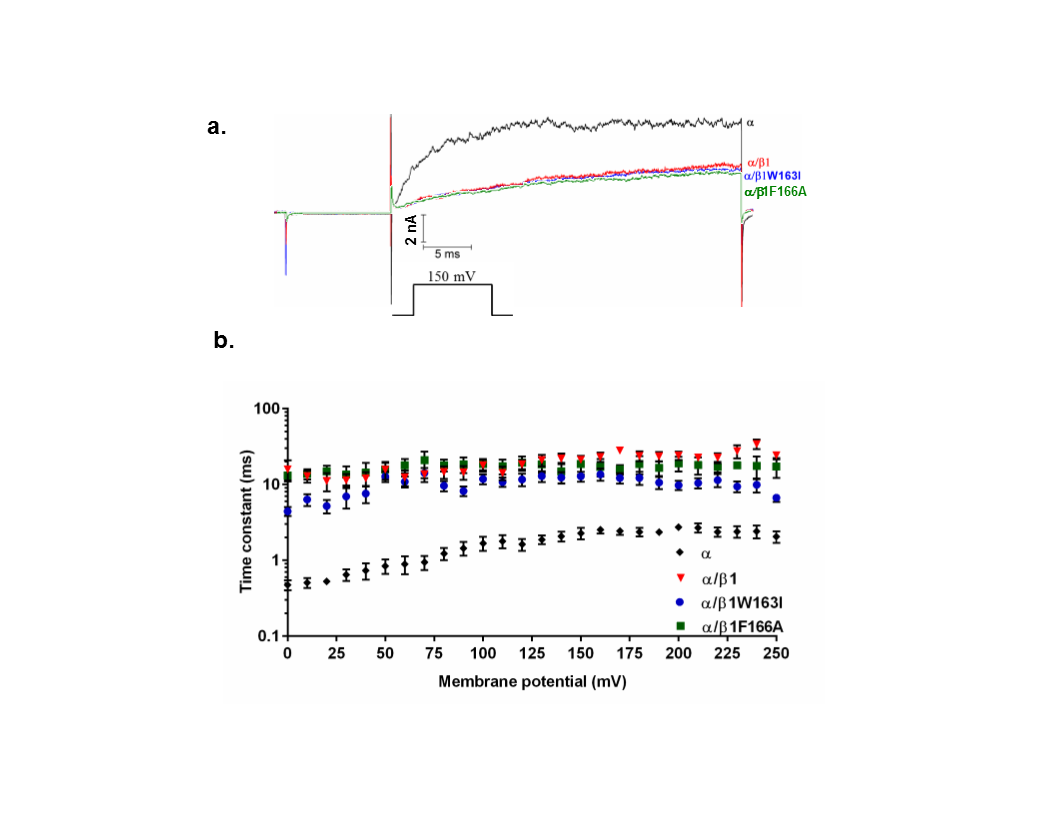
**

**Supplementary Figure S2. Effect of β1W163I and β1F166A on BK channel macroscopic kinetics in the virtual absence of Ca^2+^. (a)** Representative macroscopic current recordings at 150 mV from α channels (black traces), α/β1 channels (red traces), α/β1W163I channels (blue traces), and α/β1F166A channels (green traces). **(b)** Activation time constant (τ _Activ_) plotted against activation voltage. Symbols represent mean ± SD. α channels (⬥, n=5); α/β1 channels (▼, n=5); α/β1W163I channels (●, n=6) and α/β1F166A channels (◼, n=9)

**Supplementary Figure 3.**

**

**

**Supplementary Figure 3. E2 effect in BK channel ionic currents over time.**  Normalized currents after 5, 15, and 30 minutes of 10 μM E2 exposure. The currents of each of condition were normalized with respect to the currents evoked before the E2 stimuli in each patch (time 0) at 150 mV. Black bars: α subunit, red bars: α/β1 subunit, blue bars: α/β1W163I, green bars: α/β1F166A. Filled bars, 5 min, squared pattern bars: 15 min, dashed pattern bars, 30 min. n= >5. ***P<0.001.
