# Supplementary material for: The molecular nature of the 17β-Estradiol binding site in the voltage- and Ca2+-activated K+ (BK) channel β1 subunit
Source: Sci Rep. 2019 Jul 10;9:9965. doi: 10.1038/s41598-019-45942-1 (PMC6620312; doi:10.1038/s41598-019-45942-1)
Supplement: Supplementary file 2 — Dataset 1 [file 41598_2019_45942_MOESM2_ESM.zip › TitlepageGranadosModel_B1-E2PDBfile.docx]

**The molecular nature of the 17β-Estradiol binding site in the voltage- and Ca^2+^-activated K^+^ (BK) channel β1 subunit**

Sara T. Granados^1,2^, Karen Castillo^2^, Felipe Bravo-Moraga^2,3^, Romina V. Sepúlveda^2,3^, Willy Carrasquel-Ursulaez^2^, Maximiliano Rojas^,3^, Emerson Carmona^2^, Yenisleidy Lorenzo-Ceballos^2^, Fernando González-Nilo^2,3^, Carlos González^2^, Ramón Latorre^2,*^, Yolima P. Torres^1,*^.

^1^ Departamento de Nutrición y Bioquímica, Facultad de Ciencias, Pontificia Universidad Javeriana, Bogotá, Colombia.

^2^ Centro Interdisciplinario de Neurociencia de Valparaíso, Facultad de Ciencias, Universidad de Valparaíso, Valparaíso, Chile.

^3^ Center for Bioinformatics and Integrative Biology, Facultad de Ciencias de la Vida, Universidad Andrés Bello, Chile.
