# Supplementary figures and images for: The molecular nature of the 17β-Estradiol binding site in the voltage- and Ca2+-activated K+ (BK) channel β1 subunit
Source: Sci Rep. 2019 Jul 10;9:9965. doi: 10.1038/s41598-019-45942-1 (PMC6620312; doi:10.1038/s41598-019-45942-1)

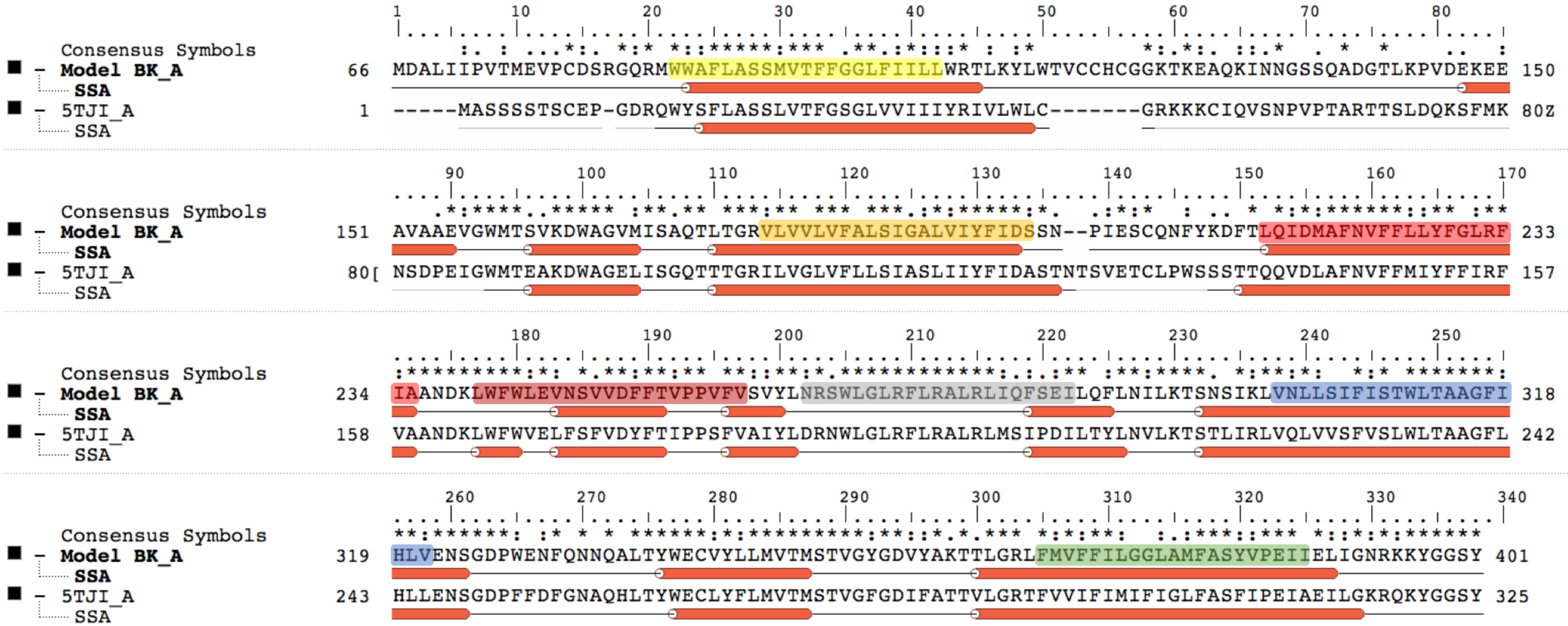

Transmembrane Segment

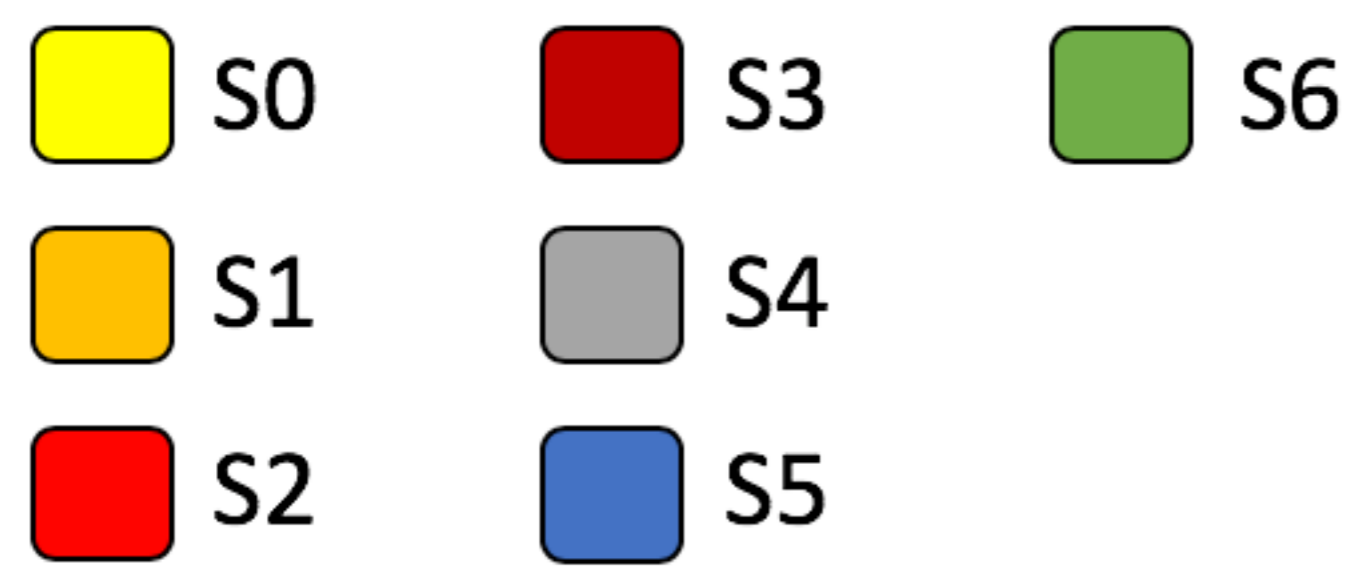

Supplement: Supplementary file 3 — Dataset 2 [file 41598_2019_45942_MOESM3_ESM.zip › BK_5TJI_Alignement.pdf]
